# The Hospital Problem

**Published:** 1922-08

**Authors:** 


### THE HOSPITAL PROBLEM.

THE Hampstead Council of Social Welfare met recently at the Town Hall under the presidency of the Mayor to discuss the Hospital Problem.

Mr. E. W. Morris, C.B.E., House Governor of the London Hospital, opened the discussion. He reminded the Council that the hospitals themselves were sick. They had been dealing with sickness for centuries, and now they themselves, who had been on full duty for a great many years, were on the danger list. He asked the Council to follow with him the history of our great hospitals, for it would bear closely on the subject before them. St. Bartholomew's, now celebrating its 800th birthday, was founded by the court jester in the reign of Henry I, in the "Smooth fields" (now Smithfields) outside the gates of London; and the first doctors and nurses were monks. St. Thomas's, named after St. Thomas à Becket, was founded by a lady who ran a ferry over the river near London Bridge and founded a Priory (most of the hospitals started with a religious movement) somewhere near where Southwark Cathedral now stands. When the Reformation came, many religious houses were closed, and amongst them these two; but the citizens appealed to the King to re-establish St. Bartholomew's, because "the miserable people lying on the footway offended every clean person with their noisome and filthy savours." They were thus herded into hospital, not so much for their own cure as for the sake of those who objected. There is then a gap right on to 1700. From 1723 to 1760 nearly every hospital in London was founded, and at the same time nearly all the well-known hospitals in the provinces; and one cannot help asking, why this great gap, and then a sudden rush for hospitals. Many reasons had been given. The plague of London, which had its greatest of all outbreaks in 1665, put everybody into a scare, and everybody was trying to do something for the sick poor because of their own dread of disease. It was not until the plague, typhus, the bad harvests about 1750 and the heavy winters (the Thames was frozen over two or three times) made such poverty in the land that sickness was rampant. One thing to notice was that the hospitals were in those days practically only shelters. It was easier to be ill in hospital than in the cold and wet outside. The patients got warmth, shelter, food and rest, and little more. Then it was not very expensive to run a hospital. There were no anæsthetics, no trained nurses, but women hired in in the night, generally drunken old widows. There was no fighting of disease. The hospital was an almshouse, and nothing more. It was quite recently, almost in the last 30 years, that the attitude of the hospital had changed. It was no longer an almshouse, but had become a fighting machine. It was interesting to note the advances made at the London Hospital during the last 25 years. In 1895 the Electrical Department was opened; in 1896 the Röntgen Rays Department (for radiography only, not for treatment); in 1899, the Finsen Light; in 1901, the Pathological Department; in 1909, the Inoculation Department, and £20,000 was given for research work; in 1910, X-ray treatment was instituted; in 1911, the Department of Research; in 1915, wards were opened for venereal diseases. If you were to wipe out all those departments, what had you left?—and it was striking to note the following record, dated 1849: "Decided to buy a microscope"!

This change of conception of a hospital as to its duty had ruined it. It was much cheaper to have an almshouse than a fighting machine against disease, and it was because of their solemn intention to take up their duty to fight disease that they found themselves where they were to-day. When a business opened a new department it opened a new earning department; when a hospital opened a new department it opened a new spending department. That spending department grew and advanced and became a more and more costly thing. During the time the speaker had been at the London Hospital the weekly cost of a bed had risen from two to five guineas. Had they anything to show for it? The death-rate had certainly come down; the average residence had greatly decreased, thus returning the wage-earner in 16 instead of 60 days and increasing the accommodation for in-patients to 23,000 a year. But the best of all tests of what a hospital was doing was the increasing number of those who never came in at all, *because of what the hospital had done*; and he believed the day was coming when our fever and smallpox hospitals would be shown as reminiscences. That was why the hospitals were where they were. It was not chiefly due to the war, as some imagined, but because of the determination of the hospital to fight. The tragedy of a starving hospital was not that you might have to close beds, but that you might cease to be a fighting machine against all that destroyed manhood and womanhood. If all that was true, had the voluntary hospital the right to exist at all? After 30 years' work at a voluntary hospital, he was ready to give up his leaning towards the voluntary system if anyone could show him a better way. But it was, in his opinion, impossible to exaggerate the tremendous merits of the voluntary system. It had taken hundreds of years to bring the spirit into a hospital that made it bad form to neglect a patient, that made the surgeon from Harley Street not only go when the telephone rang, but hurry. That spirit was not to be got in a day. It was so hard to define—so easy to see in a hospital. In any changes that might be proposed he hoped care would be taken not to interfere with this growth of the centuries. It was a very precious thing. Another merit of the voluntary hospital was its extreme elasticity. The speaker described the almost impossible tasks performed during the war, in providing at a few hours' notice accommodation for several hundred wounded men. Turning to the defects, he considered they were chiefly defects of heredity.

None of the founders of our great hospitals had foreseen any system in planting the hospitals just where they were. They put the hospitals as memorials to themselves, where they were born or where they lived. We therefore got a group of hospitals in a cluster, and they were not in the places where they were wanted. Then there was no co-ordination amongst them. He knew there were 811 cases awaiting admission to his own hospital, but he could not use St. Bart's, the Royal Free or any other hospital, and it seemed to him a monstrous thing. There was also a great waste of brains and labour amongst them. There was no standard of diets and stock medicines. Each hospital had its own. They were rival tradesmen and not fellow-soldiers. There was another foolish thing which all the hospitals did. Mr. Morris described how a patient might pass through four or five departments before reaching the surgeon's hands fo a necessary operation. And then the patient blocked a bed for three weeks whilst his wound was healing. What ought to happen was that within a week, or as soon as it was possible for him to bo moved, the patient should be moved to the outskirts of London. The cure of a bruised man would best be carried out under God's trees and not under the roof of a London hospital. In connection with the London Hospital they had two such "wards" outside the hospital, for instance, at Reigate and at Morden. A bed in the hospital cost five guineas—a bed in the ward at Reigate cost less than two. An extension of such a system would help to solve the hospital problem. Can a salaried service do its work better than a voluntary service ? In his opinion, it could not ; but the voluntary service could be so improved by introducing a system of partial contributions by patients that many failures could be put right and a sound system take the place of the present unsatisfactory one. Mr. Morris closed with the quotation : "The voluntary system is the heritage of our race, and it would be a tragedy if by our folly we threw it away."

Dr. Nathan Raw, M.P., followed. He agreed with what Mr. Morris had said as to the excellent work done in the voluntary hospitals. But we had to consider what was the best hospital system for the great mass of the community ; which was the most efficient and economical. He had had charge not only of royal infirmaries and voluntary hospitals, but of poor law infirmaries and fever hospitals, and during the war of a large military hospital in France. Having had experience of the working of all the possible kinds of hospitals, he asked : Which was the most efficient and freest from financial difficulties ? He could say unhesitatingly that the military hospital, as it was in France, was by far the best. They had a perfect system of discipline, an excellent staff, a splendidly-trained staff of hospital nurses—and unlimited means. Anything that was wanted was ordered and was got. Of course he was speaking of war-time when money was no object, and he did not say that the military hospital in peace-time would be so well equipped and so efficacious as in war-time. The efficiency of an institution depended to a great extent on finance. England was perhaps the only country in the world that had never made proper provision for hospital treatment and had left matters entirely to the charitable institutions, who had done the work so well. To-day we had 175,000 hospital beds in England. 95,000 were in poor-law institutions, 40,000 were in voluntary hospitals, 32,000 were fever hospitals, controlled by the municipalities, and over and above that great mass we had 150,000 beds for the treatment of lunatics and 14,000 beds for the treatment of tuberculosis. Who were the different authorities providing for this great mass of the sick ? They were : (1) Voluntary committees, consisting of charitably disposed persons who raised the funds for 40,000 out of the 175,000 beds ; (2) the county councils, which dealt with 150,000 lunatics' beds at the expense of the county rate ; (3) 32,000 beds in fever hospitals which were provided by the health authorities and the borough councils, and (4) the great bulk of hospital beds provided out of the rates by the poor law authorities. Was it not possible in some way to co-ordinate these various authorities, and to promote some scheme by which a much better hospital system could be provided with a saving of money ? The amount of overlapping which existed was really extraordinary, and he would have his hearers consider for a moment the misnomer of the two great systems they had before them. "Voluntary hospitals"—which were in essence hospitals for purely charitable purposes, founded, designed and endowed for the treatment of cases requiring charity ; "poor-law infirmaries"—founded in the reign of Queen Elizabeth for the purpose of dealing only with the destitute. It was contrary to law even at the present moment for any poor-law institution to deal with a case which was not destitute ; and yet the curious position to-day was that the voluntary hospitals were dealing with the well-to-do and leaving the people for whom they were really designed to a great extent to the poor law. He was glad to think that the great social progress of the last 50 years and the better general conditions that prevailed had practically wiped out destitution. Of all hospital institutions the poor law infirmary system was the most powerful. They had power to assess all property for the purposes of rates. They were here before the ordinary health authorities were in existence ; they did practically all the work of registration of births and deaths and were really the pioneers of our great municipal health system ; and now the poor-law institutions had developed entirely beyond what the law intended them to do. Instead of confining themselves to the destitute poor, they had built magnificent infirmaries, supplied with the latest equipment, and in many of them were quite capable of giving the most expert treatment. Could not more use be made of these poor-law infirmaries ? There were about 20,000 empty beds in poor-law institutions to-day. They were not being used because the destitute poor were no longer there. It had been suggested that paying patients should go into poor-law institutions, and that they should be under the care and treatment of their own medical men. That had been tried in some of the very best poor-law institutions in the north and south of England, and had failed for many reasons. First of all, according to law the medical superintendent of a poor-law infirmary was supreme. He could refuse permission for any doctor or physician to come within that institution. Then the British public had a rooted objection to being associated with the poor law in any form. For practical purposes there were only 40,000 beds available in the voluntary institutions in this country to deal with the ordinary sickness of the British public. Every one was agreed that it was absolutely inadequate. In his judgment the only thing to do was to retain the voluntary hospital system as it was, but they ought to transfer the poor law infirmary from the poor law to the municipal authority as a municipal hospital for the district; and every citizen of whatever rank should be allowed to use the municipal hospital and pay according to his means. In that way they would gain between 70,000 and 75,000 poor-law infirmary beds throughout the country. For those who were unable to pay at all, the voluntary hospitals could reserve, say, one-fourth of their beds. In conclusion, Dr. Raw regarded the voluntary hospital system as absolutely necessary, but it could not possibly continue what it was doing, for the simple reason that the great financial strain on the country and the increase in the cost of running a hospital made it impossible to raise the money required to work efficiently. If beds were kept open it would be at the cost of efficiency. Therefore he desired that the voluntary system should be carried on with some support from the local health authority and by payment by the health authority for all the cases that they treated free of charge. If a hospital had 100 beds for poor patients, it should recover the cost of these from the districts from which they came.

